# Poster Session I - A83 SUPPORTING TRANSITION IN CELIAC DISEASE: USABILITY TESTING OF A YOUTH-FOCUSED TRANSITION GUIDEBOOK

**DOI:** 10.1093/jcag/gwaf042.083

**Published:** 2026-02-13

**Authors:** E Rival, A Ramuscak, I Martincevic, A Puran, P Marcon, C M Walsh

**Affiliations:** University of Toronto Temerty Faculty of Medicine, Toronto, ON, Canada; The Hospital for Sick Children, Toronto, ON, Canada; The Hospital for Sick Children, Toronto, ON, Canada; The Hospital for Sick Children, Toronto, ON, Canada; The Hospital for Sick Children, Toronto, ON, Canada; The Hospital for Sick Children, Toronto, ON, Canada

## Abstract

**Background:**

Celiac Disease (CeD) is a chronic autoimmune condition requiring lifelong adherence to the gluten-free diet. Transitioning to adult care can be challenging for youth with CeD, as it requires developing self-management skills during a time when dietary adherence is often difficult. Little is known about how youth navigate this vulnerable period and whether tools like a guidebook can support their transition.

**Aims:**

We aimed to engage youth in evaluating and refining a Transition Guidebook for CeD through iterative usability testing.

**Methods:**

Youth with CeD aged 12 to 25, participated in usability testing of the Transition Guidebook through three iterative cycles of one-on-one, semi-structured interviews. Participants were asked about usability attributes, including content, clarity, ease of use, visual design, format and delivery-preferences. After each round, interview transcripts were analyzed through thematic coding and team discussions to identify key insights. Coding was both deductive, using published usability attributes, and inductive to capture additional emerging themes. Revisions to the guidebook were made between cycles based on participant feedback and identified usability-related themes.

**Results:**

Seventeen participants with CeD were interviewed across 3 rounds. The median age was 16 years (range 13-22), 10 (59%) were female. Several prominent themes emerged. Participants found the guidebook helpful, particularly for life-transitions like post-secondary education, and valued the sections on understanding CeD, living with CeD and adult healthcare visits. Suggestions included adopting a more empathetic tone to highlight the challenges of living with CeD, incorporating label-reading resources and providing more content on subjects involving adolescent development and independence such as travel, alcohol, cannabis and relationships. Participants emphasized the importance of using different organizational styles for text (e.g., jot notes, tables, charts) to maintain engagement and recommended adding more visual aids and coloured text to further enhance readability and interest. Revisions to the guidebook addressing participants’ suggestions in the first two rounds were well received in subsequent rounds.

**Conclusions:**

Our findings highlight the value of a participatory, iterative process to develop a guidebook ensuring it effectively supports youths’ transition to adult care for CeD. Ongoing feedback will inform further refinements to enhance usability and relevance.

**Funding Agencies:**

None

